# The Neuroprotective Effects of Flavonoid Fisetin against Corticosterone-Induced Cell Death through Modulation of ERK, p38, and PI3K/Akt/FOXO3a-Dependent Pathways in PC12 Cells

**DOI:** 10.3390/pharmaceutics15102376

**Published:** 2023-09-23

**Authors:** Pei-Rong Chang, Je-Wen Liou, Pei-Yi Chen, Wan-Yun Gao, Chia-Ling Wu, Ming-Jiuan Wu, Jui-Hung Yen

**Affiliations:** 1 Department of Molecular Biology and Human Genetics, Tzu Chi University, Hualien 970374, Taiwan; 880802candy@gmail.com (P.-R.C.); pyc571@gmail.com (P.-Y.C.); 2 Department of Biochemistry, School of Medicine, Tzu Chi University, Hualien 970374, Taiwan; jwliou@mail.tcu.edu.tw; 3 Laboratory of Medical Genetics, Genetic Counseling Center, Hualien Tzu Chi Hospital, Buddhist Tzu Chi Medical Foundation, Hualien 970374, Taiwan; 106726102@gms.tcu.edu.tw; 4 Institute of Medical Sciences, Tzu Chi University, Hualien 970374, Taiwan; 108353105@gms.tcu.edu.tw; 5 Department of Biotechnology, Chia-Nan University of Pharmacy and Science, Tainan 717301, Taiwan; mingjiuanwu@gmail.com

**Keywords:** hypothalamic–pituitary–adrenal axis, corticosterone, fisetin, ERK, PI3K/Akt, FOXO3a

## Abstract

The overactive hypothalamic–pituitary–adrenal (HPA) axis is believed to trigger the overproduction of corticosterone, leading to neurotoxicity in the brain. Fisetin is a flavonoid commonly found in fruits and vegetables. It has been suggested to possess various biological activities, including antioxidant, anti-inflammatory, and neuroprotective effects. This study aims to explore the potential neuroprotective properties of fisetin against corticosterone-induced cell death and its underlying molecular mechanism in PC12 cells. Our results indicate that fisetin, at concentrations ranging from 5 to 40 μM, significantly protected PC12 cells against corticosterone-induced cell death. Fisetin effectively reduced the corticosterone-mediated generation of reactive oxygen species (ROS) in PC12 cells. Fisetin treatments also showed potential in inhibiting the corticosterone-induced apoptosis of PC12 cells. Moreover, inhibitors targeting MAPK/ERK kinase 1/2 (MEK1/2), p38 MAPK, and phosphatidylinositol 3-kinase (PI3K) were found to significantly block the increase in cell viability induced by fisetin in corticosterone-treated cells. Consistently, fisetin enhanced the phosphorylation levels of ERK, p38, Akt, and c-AMP response element-binding protein (CREB) in PC12 cells. Additionally, it was found that the diminished levels of p-CREB and p-ERK by corticosterone can be restored by fisetin treatment. Furthermore, the investigation of crosstalk between ERK and CREB revealed that p-CREB activation by fisetin occurred through the ERK-independent pathway. Moreover, we demonstrated that fisetin effectively counteracted the corticosterone-induced nuclear accumulation of FOXO3a, an apoptosis-triggering transcription factor, and concurrently promoted FOXO3a phosphorylation and its subsequent cytoplasmic localization through the PI3K/Akt pathway. In conclusion, our findings indicate that fisetin exerts its neuroprotective effect against corticosterone-induced cell death by modulating ERK, p38, and the PI3K/Akt/FOXO3a-dependent pathways in PC12 cells. Fisetin emerges as a promising phytochemical for neuroprotection.

## 1. Introduction

Stress can trigger the release of corticosteroids, which exert widespread effects in the central nervous system. Prolonged exposure to stress and elevated corticosteroid levels may lead to neuropathology, cognitive impairment, psychiatric conditions, and neurodegenerative disorders [1]. The hypothalamic–pituitary–adrenal (HPA) axis, a hypothetical construct comprising the hypothalamus, pituitary gland, and adrenal gland, becomes activated in response to stress [2]. This activation is primarily instigated by corticotropin-releasing hormone (CRH) and vasopressin (VAP) secreted by the hypothalamus. In response, the pituitary gland releases adrenocorticotropic hormone (ACTH), which then stimulates the adrenal cortex to generate glucocorticoids [3]. These glucocorticoids serve as crucial stress hormones, overseeing a diverse array of physiological processes including metabolism, immune function, skeletal growth, cardiovascular function, reproduction, and cognition [4]. The regulation of the HPA axis is a promising avenue for the development of therapeutic agents for stress-related mental dysfunction or major depressive disorders [5,6].

Under normal conditions, blood glucocorticoid levels are tightly regulated via negative feedback on the HPA axis. This negative feedback mechanism involves the inhibition of CRH and ACTH production, facilitated by the action of glucocorticoids on their receptors within the hypothalamus and pituitary gland. However, when individuals experience chronic stress, their blood glucocorticoid levels may escalate, causing dysfunction in the HPA axis and potentially exacerbating depression [2,4,7]. It has been reported that the hyperfunction of the HPA axis leads to a sustained increase in the level of corticosterone, a type of glucocorticoid produced by the adrenal cortex [8]. Furthermore, excessive corticosterone production has been observed to reduce the release of serotonin (5-hydroxytryptamine, 5-HT), a neurotransmitter that plays crucial roles in mood regulation [9]. The cellular mechanisms underlying corticosterone-induced neurotoxicity involve oxidative stress characterized by the overproduction of reactive oxygen species (ROS) [10] as well as inflammatory responses [11]. Corticosterone has been reported to induce neuronal cell death and diminish neurogenesis in the hippocampal region [12,13]. These processes contribute to neuronal apoptosis and neurological alterations, potentially linking to the pathological changes observed in the brain.

Multiple molecular signaling mechanisms of anti-oxidation and cell survival are suggested to be involved in neuroprotective effects, including the mitogen-activated protein kinase (MAPK) pathways such as extracellular signal-regulated kinases (ERK), c-Jun amino-terminal kinases (JNK), and p38/MAPK, as well as the phosphoinositide 3-kinase (PI3K)/Akt pathway [14,15,16]. Previous studies demonstrated that the MAPK/ERK pathway plays a critical role in a variety of neuronal cell responses [17]. Upon the activation of ERK, it regulates the activity of various transcription factors, including the cAMP-response element binding protein (CREB), through phosphorylation [18]. When CREB is activated, it enhances the transcriptional activity of the cAMP response element, subsequently promoting the expression of genes are responsible for neuronal cell survival, growth, proliferation, plasticity, and differentiation [19,20,21]. The PI3K/Akt pathway plays a significant role in governing diverse neurological processes, including neuronal cell survival, differentiation, and synaptogenesis [22,23]. The modulation of the PI3K/Akt-forkhead box O3 (FOXO3a) pathway holds crucial importance in regulating neuronal cell survival. FOXO3a, a member of the forkhead box protein O (FOXO) family, functions as a critical transcription factor that regulates cell proliferation, apoptosis, oxidative stress, and DNA damage [24]. Upon the activation of the PI3K/Akt kinase signaling, there is a subsequent promotion of FOXO3a phosphorylation, leading to the translocation of the FOXO3a protein from the nucleus to the cytoplasm for degradation [25,26]. The down-regulation or inactivation of FOXO3a, achieved by reducing its nuclear retention within cells, hinders apoptosis and promotes cell survival.

The investigation of effective agents with antioxidant or anti-inflammatory activities from natural phytochemicals is emerging as a prominent strategy for the development of neuroprotective agents [27,28]. Fisetin (3,3′,4′,7-tetrahydroxyflavone), a dietary flavonoid, is widely present in fruits and vegetables, including strawberries, persimmons, grapes, onions, cucumbers, and nuts [29]. Fisetin has been reported to exhibit antioxidative, anti-inflammatory, anti-tumorigenic, anti-diabetic, and cardioprotective properties [29,30,31,32]. It has demonstrated neuroprotective effects by suppressing oxidative stress and neuronal cell death, promoting neuronal survival, and enhancing learning and memory in various neurodegenerative diseases [33,34,35,36]. Additionally, fisetin has been found to alleviate depressive-like behaviors induced by lipopolysaccharide (LPS) through the inhibition of inflammatory responses [37] and the reduction in ROS generation in animal models [38]. Moreover, fisetin has shown to increase the levels of phosphorylated tropomyosin receptor kinase B (TrkB) and exhibit an antidepressant effect by activating the TrkB signaling pathway in mice [39].

The aim of this study was to explore the potential neuroprotective attributes of fisetin against corticosterone-induced neurotoxicity in a rat pheochromocytoma PC12 cell model. Additionally, our objective was to uncover the underlying molecular mechanism involved.

## 2. Materials and Methods

### 2.1. Chemicals

Fisetin (3,3′,4′,7-tetrahydroxyflavone), dimethyl sulfoxide (DMSO), poly-L-lysine, thiazoly blue tetrazolium bromide (MTT), nonessential amino acids (NEAAs), RPMI-1640 medium, and other chemicals were purchased from Sigma–Aldrich (St. Louis, MO, USA) unless otherwise indicated. Corticosterone and SP600125 were purchased from Enzo Life Sciences (Farmingdale, New York, NY, USA). Horse serum (HS) and fetal bovine serum (FBS) were purchased from Thermo Fisher Scientific (Rockford, IL, USA). U0126, SB203580 and LY294002 were purchased from Promega (Madison, WI, USA).

### 2.2. Cell Culture

The PC12 cells were obtained from the Bioresource Collection and Research Center (BCRC, Hsinchu, Taiwan). Cells were maintained in RPMI-1640 medium composed of 10% heat-inactivated HS, 5% FBS, and 1% NEAA. The cells were cultured in a 5% CO_2_ incubator at 37 °C.

### 2.3. Compounds Treatment

For the treatment of compounds, PC12 cells were seeded at a density of 2 × 10^5^ cells/mL in 24-well plates using a low-serum RPMI medium (1% HS, 0.5% FBS, and 1% NEAA). The cells were then treated with the specified compounds for subsequent analysis. In the case of corticosterone treatment, the cells were exposed to either the vehicle or corticosterone (100–800 μM) for 24 h. In experiments involving the co-treatment of corticosterone and fisetin, the cells were subjected to corticosterone treatment (300 µM) in the absence or presence of fisetin, which was dissolved in a vehicle containing 0.1% DMSO, at the indicated concentrations. For the treatment involving kinase inhibitors, the cells were pre-treated with various inhibitors: MAPK/ERK kinase1/2 (MEK1/2) inhibitor U0126 (10 μM), JNK inhibitor SP600125 (10 μM), p38 MAPK inhibitor SB203580 (10 μM), and PI3K inhibitor LY294002 (40 μM) for 30 min. Subsequently, the cells were treated with corticosterone (300 µM) in the absence or presence of fisetin (40 μM) for an additional 24 h.

### 2.4. Analysis of Cell Viability

The MTT assay was employed to assess cell viability as previously described [40]. Following the compounds treatment, the cells were exposed to an MTT reagent (1 mg/mL) and incubated for 3 h at 37 °C. After the incubation, the cells were collected through centrifugation at 13,000 rpm for 5 min. The supernatant was then discarded, and the resulting purple crystals were dissolved using DMSO. Cell viability was assessed by measuring the absorbance at 550 nm.

### 2.5. Analysis of ROS Generation

The ROS production was measured by flow cytometry as previously described [36]. PC12 cells were seeded onto poly-L-lysine-coated 6-well plates and cultured in normal serum medium for 24 h. The medium was subsequently removed and replaced with low-serum RPMI medium. Then, 5 μM of 2′, 7′-dichlorodihydrofluorescein diacetate (H_2_DCFDA) (Thermo Fisher Scientific) was introduced to the cells and incubated at 37 °C for 30 min in a dark environment. After the incubation, the cells were subjected to treatment with corticosterone (300 µM), either in the absence or presence of fisetin, for a 24 h duration. Subsequently, the cells were treated with 1× trypsin–EDTA for 2 min to detach adherent cells, facilitating the analysis of ROS. The level of intracellular ROS was assessed by detecting the fluorescence. Three independent samples, each containing 10,000 cells, were analyzed using the Beckman CytoFLEX Flow Cytometer equipped with a FITC emission filter (Beckman Coulter, Inc., Brea, CA, USA). The resulting data were expressed as the relative percentage of the geometric mean fluorescence intensity.

### 2.6. Analysis of Cell Apoptosis by Fluorescent Microscopy

PC12 cells were seeded onto poly-L-lysine coated coverslips and cultured in normal serum medium for 24 h. On the following day, the culture medium was replaced with low-serum RPMI medium, and the cells were treated with corticosterone (300 µM) in the absence or presence of fisetin (40 μM) for 24 h. After incubation, the coverslips were carefully lifted and placed onto slides. Subsequently, they were mounted using 20 µL of VECTASHIELD^®^ Vibrance™ Antifade Mounting Medium with 4′,6-diamidino-2-phenylindole (DAPI) (Vector Laboratories, Burlingame, CA, USA). The images were then observed and captured using a Nikon Ti2-E inverted fluorescent microscope along with the NIS-Element imaging software Version 5.5 (Nikon Instruments, Tokyo, Japan). The resulting images were subjected to analysis using a tally counter. The apoptotic cells were analyzed across 35 randomly chosen microscopic fields, with each group containing over 600 cells.

### 2.7. Analysis of Cell Apoptosis by Flow Cytometry

The cell apoptosis was measured by flow cytometry as previously described [40]. To determine the proportion of apoptotic cells, cells were treated with corticosterone (300 µM) in the absence or presence of fisetin (40 μM) for 24 h. After incubation, cells were washed in ice-cold PBS and the apoptotic cells were analyzed by an Annexin V-FITC Apoptosis Detection Kit (Strong Biotech, Taipei, Taiwan) according to the manufacturer’s instructions. The population of apoptotic cells was detected using a Beckman CytoFLEX Flow Cytometer (Beckman Coulter).

### 2.8. Proteins Preparation and Western Blot Analysis

For the preparation of total cellular proteins, the cell lysates were isolated using RIPA buffer (Thermo Fisher Scientific) containing 1× Protease Inhibitor Single-Use Cocktail (Thermo Fisher Scientific) and PhosphataseArrest^TM^ II (G-Biosciences, Saint Louis, MO, USA). For the preparation of nuclear proteins, the protein lysates were extracted by Nuclear Extract Kit (Active Motif, Carlsbad, CA, USA) according to the manufacturer’s instruction. The protein concentration was determined by Bio-Rad Bradford protein assay reagents. Equal amounts of protein lysates were separated by 8%, 10%, or 12% SDS-PAGE and transferred onto a PolyScreen PVDF Transfer Membrane (Cytiva, Buckinghamshire, UK). The membranes were incubated with specific primary antibodies for rat proteins: Phospho-p44/42 MAPK kinase (Thr202/Tyr204), p44/42 MAPK kinase (Thr202/Tyr204), Phospho-p38 MAPK (Thr180/Tyr182), p38 MAPK, Phospho-Akt (Ser473), Akt, Phospho-CREB (Ser133), CREB, Phospho-FOXO3a, and FOXO3a (Cell Signaling Technology, Danvers, MA, USA); α-Tubulin and HDAC2 (GeneTex, Irvine, CA, USA); and actin (Thermo Fisher Scientific). The membranes were then incubated with horseradish peroxidase (HRP)-conjugated goat anti-mouse (Cell Signaling Technology) or anti-rabbit (GeneTex) IgG secondary antibodies. The blots were then rinsed with Amersham ECL^TM^ Prime Western blotting detection reagent, and the chemiluminescent protein signals were detected on Amersham Hyperfilm™ ECL (Cytiva).

### 2.9. Transfection of FOXO3a-GFP Expression Plasmid

PC12 cells were seeded on poly-L-lysine coated coverslip and cultured in a normal serum medium for 24 h. For plasmid transfection, cells were transfected with the pCMV6-AC-FOXO3-GFP plasmid (OriGene Technologies, Rockville, MD, USA) using Lipofectamine 2000 Transfection Reagent (Thermo Fisher Scientific). Following a 4 h transfection period, the medium supplemented with 20% HS, 10% FBS, and 1% NEAA was added and incubated for 24 h. Subsequently, the medium was replaced with low-serum RPMI medium, and the cells were treated with vehicle or fisetin (40 μM) for an additional 24 h. After the treatments, the coverslips containing the cells were carefully collected and positioned on the slides. VECTASHIELD^®^ Vibrance™ Antifade Mounting Medium was applied to the samples for mounting. Imaging was conducted using a Nikon Ti2-E inverted fluorescent microscopy. The images were captured and documented using the NIS-Element imaging software.

### 2.10. Statistical Analysis

The experiments were conducted independently on a minimum of three occasions, and within each experiment, the procedures were replicated three times. The data are presented as mean ± standard deviation (SD). Graph Pad Prism (version 9) (Graph Pad Software Inc., San Diego, CA, USA) was used to analyze the statistical significance. For comparisons involving two groups, Student’s *t*-test was utilized. When multiple groups were being compared, a one-way ANOVA followed by Tukey’s post hoc test was employed. A *p*-value of less than 0.05 was considered statistically significant.

## 3. Results

### 3.1. Effects of Fisetin on Cell Viability in Corticosterone-Treated PC12 Cells

**Figure 1:**
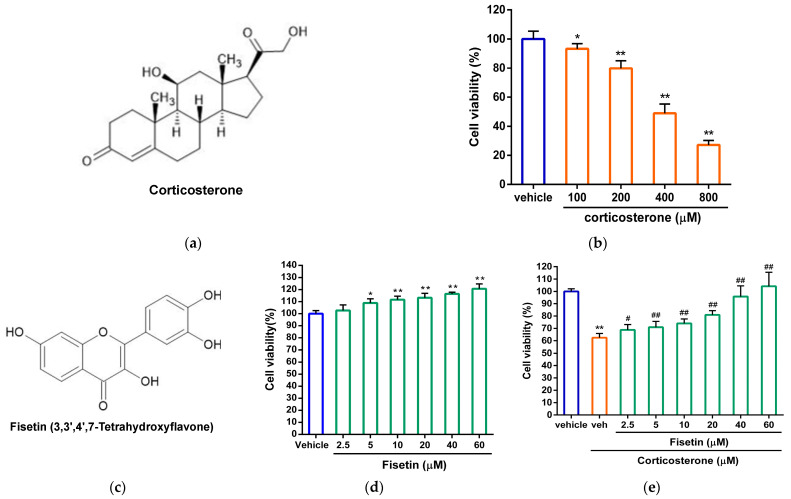
The effect of fisetin on cell viability in corticosterone-treated PC12 cells. (**a**) The chemical structure of corticosterone. (**b**) PC12 cells were treated with vehicle (0.1% DMSO) or corticosterone (100, 200, 400, and 800 µM) for 24 h. (**c**) The chemical structure of fisetin (3,3′,4′,7-tetrahydroxyflavone). (**d**) PC12 cells were treated with vehicle or fisetin (2.5, 5, 10, 20, 40, and 60 μM) for 24 h. (**e**) PC12 cells were treated with vehicle or corticosterone (300 µM) in the absence (veh) or presence of fisetin (2.5, 5, 10, 20, 40, and 60 μM) for 24 h. Cell viability was measured by MTT assay. The data represent the mean ± SD of three independent experiments. * *p* < 0.05 and ** *p* < 0.01 represent significant differences compared to the vehicle-treated group. # *p* < 0.05 and ## *p* < 0.01 represent significant differences compared to the corticosterone alone (veh)-treated group.

To confirm the cytotoxic impact of corticosterone (depicted in Figure 1a), PC12 cells were subjected to treatment with either the vehicle (0.1% DMSO) or varying concentrations of corticosterone (ranging from 100 to 800 µM) for a duration of 24 h. As shown in Figure 1b, the treatment with corticosterone led to a significant reduction in cell viability: 94.73 ± 4.20% (*p* < 0.05), 82.56 ± 5.99% (*p* < 0.01), 50.76 ± 7.17% (*p* < 0.01), and 27.12 ± 3.03% (*p* < 0.01), respectively, compared to the group treated solely with the vehicle (100.00 ± 5.40%). These data indicated that corticosterone is capable of inducing cell death in PC12 cells in a dose-dependent manner. To investigate the impact of fisetin (depicted in Figure 1c) on the viability of PC12 cells, the cells were treated with either the vehicle or various concentrations of fisetin (ranging from 2.5 to 60 μM). The results, as shown in Figure 1d, revealed that fisetin treatment did not induce any cytotoxic effects on PC12 cells. To further explore the protective effects of fisetin against corticosterone-induced cell death, PC12 cells were subjected to a 24 h exposure to corticosterone (300 μM), either in the presence or absence of fisetin. As shown in Figure 1e, the treatment with corticosterone resulted in a significant reduction in the cell viability of PC12 cells to 62.40 ± 3.50%. Conversely, the co-administration of fisetin (at concentrations of 2.5, 5, 10, 20, 40, and 60 μM) markedly elevated the cell viability to 68.75 ± 4.37%, 70.98 ± 4.62%, 73.97 ± 3.68%, 80.93 ± 3.39%, 95.83 ± 8.72%, and 104.21 ± 11.33%, respectively, in comparison to cells only treated with the vehicle (100.00 ± 2.17%). These findings reveal the significant protective effect of fisetin in guarding PC12 cells against cell death induced by corticosterone.

### 3.2. Effects of Fisetin on ROS Production in Corticosterone-Treated PC12 Cells

**Figure 2:**
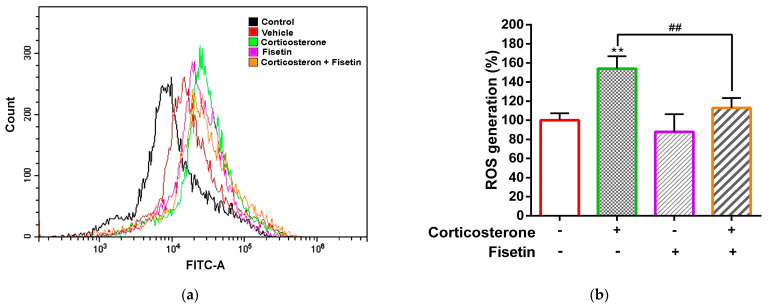
The effect of fisetin on the production of ROS in PC12 cells treated with corticosterone. PC12 cells were cultured in a poly-L-lysine-coated 6-well plate and pre-incubated with H_2_DCFDA (5 μM) at 37 °C for 30 min in the dark. Following incubation, the cells were treated with corticosterone (300 µM), either in the absence or presence of fisetin (40 µM), for a duration of 24 h. The level of ROS was quantified by flow cytometry analysis. (**a**) A representative histogram featuring the control group (the black line), cells subjected to vehicle treatment (the red line), cells treated with corticosterone (the green line), cells treated with fisetin (the pink line), and cells co-treated with both corticosterone and fisetin (the orange line). (**b**) Quantification of ROS levels. The data represent the mean ± SD of three independent experiments. ** *p* < 0.01 represents significant differences compared to vehicle-treated group. ## *p* < 0.01 represent significant differences compared to corticosterone alone-treated group.

Oxidative stress contributes to numerous neurodegenerative disorders, and the generation of ROS is a critical indicator of this process. Previous studies have reported that corticosterone can induce cytotoxicity by elevating cellular ROS levels [41]. Hence, we proceeded to investigate whether fisetin possesses the ability to mitigate corticosterone-induced cell death in PC12 cells through the reduction in ROS production. As shown in Figure 2a,b, corticosterone increased cellular ROS levels by 154.03 ± 12.92% (*p* < 0.01) compared to the vehicle group (100.00 ± 7.19%). Co-treatment with fisetin significantly reduced measured ROS levels in corticosterone-treated cells to 112.83 ± 10.51% (*p* < 0.01). These findings suggest that fisetin exhibits the function to mitigate corticosterone-induced ROS production, thereby offering protection to neuronal cells against cell death.

### 3.3. Effects of Fisetin on Cell Apoptosis in Corticosterone-Treated PC12 Cells

**Figure 3:**
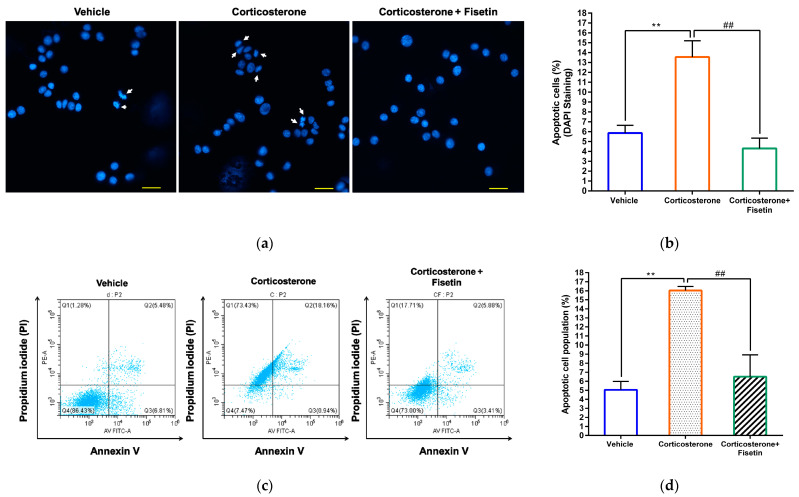
The effect of fisetin on cell apoptosis in corticosterone-treated PC12 cells. PC12 cells were treated with corticosterone (300 μM) in the absence or presence of fisetin (40 μM) for 24 h. After incubation, the cells were further incubated with DAPI. The stained cells were observed and photographed using fluorescence microscopy. (**a**) The nuclei were stained with DAPI (blue). A representative image is presented, with an arrow indicating apoptotic cells, and a scale bar measuring 20 μm. (**b**) Quantification of apoptotic cells. Apoptotic cells were quantified across 35 randomly selected fields from three independent replicates. The cell counts for vehicle-treated cells (*n* = 805), corticosterone-treated cells (*n* = 760), and cells co-treated with corticosterone and fisetin (*n* = 658) were calculated. (**c**) The cell apoptosis was assessed using flow cytometric analysis. A representative histogram illustrating cell apoptosis is shown. (**d**) The percentage of the apoptotic cell population was quantified. The data represent the mean ± SD of three independent experiments. ** *p* < 0.01 indicates significant differences compared to the vehicle-treated group. ## *p* < 0.01 indicates significant differences compared to the corticosterone alone-treated group.

Previous studies have indicated that corticosterone can induce apoptosis in neuronal cells [42]. As indicated in the previous section, fisetin was able to mitigate corticosterone-induced ROS production. This function may contribute to the inhibition of cellular apoptosis. Therefore, we conducted further investigations to explore the protective effect of fisetin against corticosterone-induced apoptosis in PC12 cells. As shown in Figure 3a,b, the DAPI fluorescent staining of cells indeed revealed that corticosterone increased the percentage of apoptotic cells to 13.55 ± 1.65% (*p* < 0.01), as compared to the vehicle-treated group (5.87 ± 0.78%). On the other hand, co-treatment with fisetin significantly reduced the occurrence of cell apoptosis to 5.83 ± 1.40% (*p* < 0.01). The flow cytometric analysis of Annexin V/PI-stained cells confirmed that fisetin treatment significantly reduced the proportion of cells undergoing corticosterone-induced apoptosis, decreasing from approximately 16% (corticosterone alone treatment) to around 6% (*p* < 0.01) (Figure 3c,d). These findings provide further support for the ability of fisetin to inhibit corticosterone-induced neuronal cell apoptosis.

### 3.4. Involvement of MAPK/ERK, p38 MAPK and PI3/Akt Pathways in the Cytoprotective Effect of Fisetin against Corticosterone-Induced Cell Death

**Figure 4:**
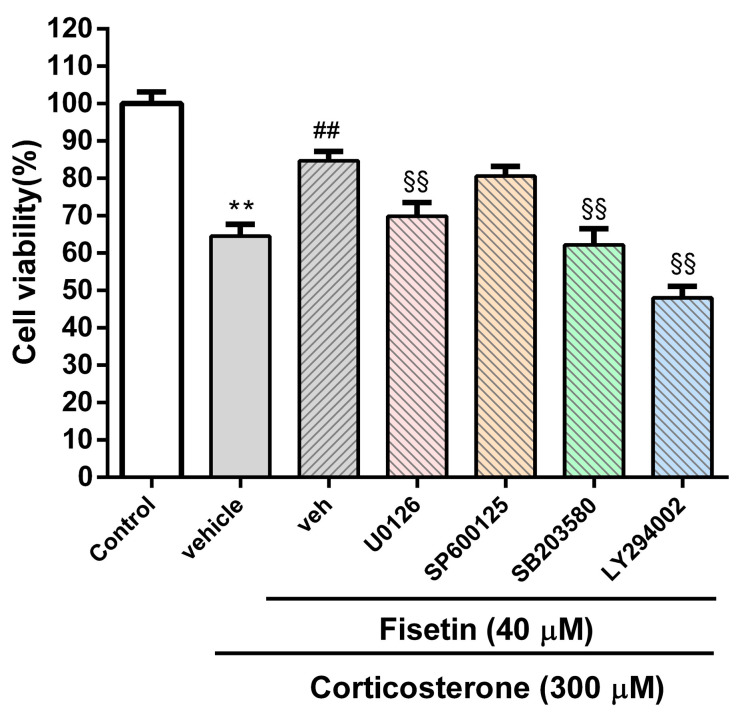
The kinase signaling pathways involved in fisetin-mediated neuroprotection in corticosterone-treated PC12 cells. Cells were pretreated with inhibitors, including U0126 (10 μM), SP600125 (10 μM), SB203580 (10 μM), and LY294002 (40 μM), respectively, for 30 min. Subsequently, the cells were exposed to corticosterone (300 µM) in the absence or presence of fisetin (40 μM) for 24 h, and cell viability was assessed using the MTT assay. The presented data represent the mean ± SD of three independent experiments. ** *p* < 0.01 indicates significant differences compared to the control group. ## *p* < 0.01 indicates significant differences compared to the corticosterone alone-treated (vehicle) group. ^§§^ *p* < 0.01 indicates significant differences compared to the inhibitor-untreated (veh) group.

To further elucidate the signaling pathways underlying the neuroprotective effect of fisetin against corticosterone-induced cell death, we employed specific kinase inhibitors, including U0126, SP600125, SB203580, and LY294002, for investigations. As shown in Figure 4, the restoration of cell viability in PC12 cells treated with corticosterone, mediated by fisetin, was observed to be less effective when specific inhibitors U0126, SB203580, and LY294002 were introduced (*p* < 0.01), strongly suggesting the potential involvement of the ERK, p38 MAPK, and PI3/Akt pathways in the cytoprotective effect of fisetin against corticosterone-induced cell death.

**Figure 5:**
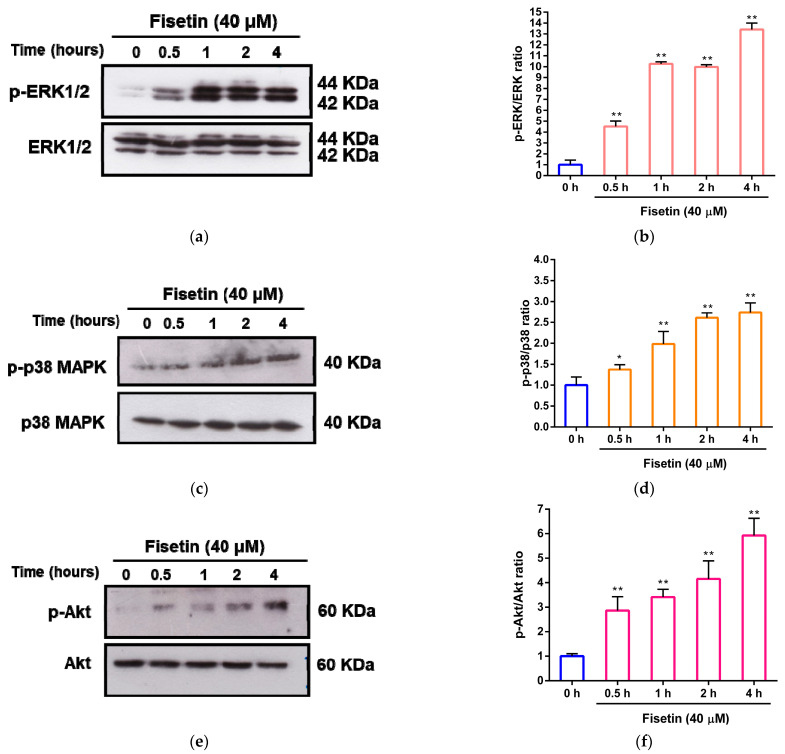
Fisetin enhanced the phosphorylation of ERK, p38, and Akt in PC12 cells. PC12 cells were treated with fisetin (40 μM) for 0.5, 1, 2, and 4 h. (**a**) p-ERK and ERK; (**c**) p-p38 and p38; (**e**) p-Akt and Akt proteins were measured by Western blot analysis, and a representative blot is shown. Quantitative analysis of normalized intensity of (**b**) p-ERK versus ERK, (**d**) p-p38 versus p38, (**f**) p-Akt versus Akt. The data represent the mean ± SD of three independent experiments. * *p* < 0.05 and ** *p* < 0.01 indicate significant differences compared to 0 h group.

Additionally, we evaluated the activation status of key signaling molecules, namely ERK, p38 MAPK, and Akt, following treatment with fisetin using Western blot analysis. The results demonstrated that upon treatment with fisetin (40 μM) for 0, 0.5, 1, 2, and 4 h, the protein ratio of phospho-ERK1/2 (p-ERK) to ERK1/2 increased from 1.00 ± 0.42-fold to 4.51 ± 0.51-fold (*p* < 0.01), 10.26 ± 0.19-fold (*p* < 0.01), 9.98 ± 0.20-fold (*p* < 0.01), and 13.41 ± 0.59-fold (*p* < 0.01), respectively, compared to the 0 h group. (Figure 5a,b). Similarly, the protein ratio of phospho-p38 (p-p38) to p38 demonstrated significant increases due to fisetin treatment at 0, 0.5, 1, 2, and 4 h, increasing from 1.00 ± 0.19-fold to 1.37 ± 0.12-fold (*p* < 0.05), 1.98 ± 0.30-fold (*p* < 0.01), 2.61 ± 0.12-fold (*p* < 0.01), and 2.74 ± 0.23-fold (*p* < 0.01), respectively (Figure 5c,d). Notably, fisetin also significantly increased the protein ratio of phospho-Akt (p-Akt) to Akt from 1.00 ± 0.11-fold to 2.87 ± 0.56-fold (*p* < 0.01), 3.41 ± 0.32-fold (*p* < 0.01), 4.15 ± 0.74-fold (*p* < 0.01), and 5.92 ± 0.70-fold (*p* < 0.01), respectively, over the 0.5~4 h interval when compared to the 0 h group (Figure 5e,f). Collectively, these findings strongly indicate that fisetin might augment cell survival by triggering the activation of the ERK, p38, and PI3K/Akt pathways, thereby acting as a defense mechanism against corticosterone-induced cell death in PC12 cells. 

### 3.5. Effects of Fisetin on CREB Activation in Corticosterone-Treated PC12 Cells

**Figure 6:**
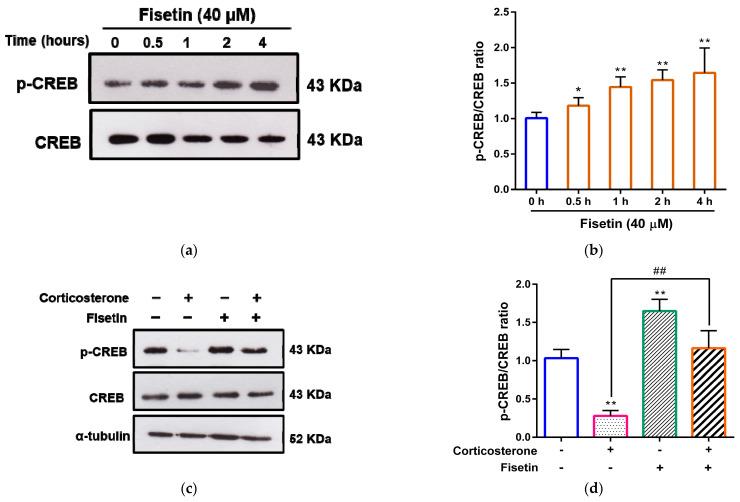
The effect of fisetin on the corticosterone-mediated inactivation of CREB. PC12 cells were treated with fisetin (40 μM) for 0, 0.5, 1, 2, and 4 h. (**a**) p-CREB and CREB proteins were measured by Western blot analysis, and a representative blot is shown. (**b**) Quantitative analysis of normalized intensity of p-CREB versus CREB. The data represent the mean ± SD of three independent experiments. * *p* < 0.05 and ** *p* < 0.01 indicate significant differences compared to the 0 h group. PC12 cells were treated with corticosterone (300 μM) in the absence or presence of fisetin (40 μM) for 24 h. (**c**) p-CREB, CREB, and α-tubulin proteins were measured by Western blot analysis, and a representative blot is shown. (**d**) Quantitative analysis of normalized intensity of p-CREB versus CREB. The data represent the mean ± SD of three independent experiments. ** *p* < 0.01 indicates significant differences compared to the vehicle-treated group. ## *p* < 0.01 indicates significant differences compared to the corticosterone alone-treated group.

Previous studies have highlighted the importance of CREB as a pivotal mediator in signal transduction pathways associating with cellular survival and mechanisms [43,44]. To investigate the possible involvement of CREB in the protective effects of fisetin in corticosterone-treated cells, we employed Western blot analysis to determine whether fisetin has the capacity to elevate the level of phospho-CREB proteins. As shown in Figure 6a,b, fisetin (40 μM) demonstrated a significant increase in the ratio of phospho-CREB (p-CREB) to CREB, rising from 1.00 ± 0.08-fold to 1.18 ± 0.11-fold (*p* < 0.05), 1.44 ± 0.14-fold (*p* < 0.01), 1.54 ± 0.14-fold (*p* < 0.01), and 1.64 ± 0.35-fold (*p* < 0.01), respectively, when compared to the 0 h group. Furthermore, to substantiate the effects of fisetin and corticosterone on phospho-CREB levels, our findings revealed that the group treated with corticosterone alone showed a reduction in phospho-CREB levels by approximately 0.28-fold (*p* < 0.01) when compared to the vehicle-treated group. In contrast, cells treated solely with fisetin demonstrated an increase in phospho-CREB levels by approximately 1.65-fold (*p* < 0.01). In the co-treatment scenario involving both corticosterone and fisetin, fisetin effectively counteracted the corticosterone-induced reduction in phospho-CREB levels from approximately 0.28-fold to 1.16-fold (*p* < 0.01) in PC12 cells (Figure 6c,d). The cumulative findings suggest that fisetin could potentially reverse the CREB inactivation induced by corticosterone in PC12 cells.

**Figure 7:**
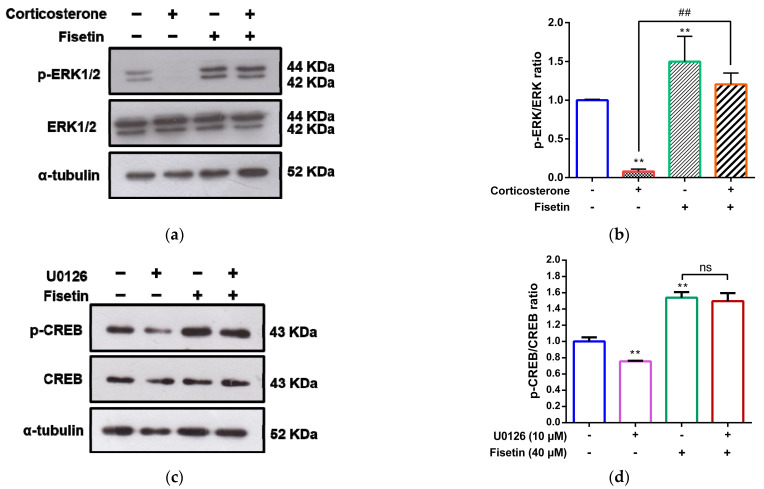
The impact of fisetin on crosstalk between the ERK pathway and CREB activation in corticosterone-treated PC12 cells. PC12 cells were treated with corticosterone (300 μM) in the absence or presence of fisetin (40 μM) for 24 h. (**a**) p-ERK, ERK, and α-tubulin proteins were measured by Western blot analysis, and a representative blot is shown. (**b**) Quantitative analysis of normalized intensity of p-ERK versus ERK. The data represent the mean ± SD of three independent experiments. ** *p* < 0.01 indicates significant differences compared to the vehicle-treated group. ## *p* < 0.01 indicates significant differences compared to the corticosterone alone-treated group. PC12 cells were pre-treated with U0126 (10 μM) for 30 min, then treated fisetin (40 μM) for 24 h. (**c**) p-CREB, CREB and α-tubulin proteins were measured by Western blot analysis, and a representative blot is shown. (**d**) Quantitative analysis of the normalized intensity of p-CREB versus CREB. The data represent the mean ± SD of three independent experiments. ** *p* < 0.01 indicates statistically significant differences compared to the vehicle-treated group. “ns” indicates no statistically significant differences.

We further investigated the potential crosstalk between the ERK pathway and CREB activation. Corticosterone prominently resulted in a reduction in phospho-ERK levels by approximately 0.08-fold, while fisetin alone significantly elevated phospho-ERK levels by approximately 1.50-fold compared to the vehicle-treated group in PC12 cells (Figure 7a,b). Importantly, in the co-treatment scenario involving both corticosterone and fisetin, fisetin effectively reversed the corticosterone-induced decrease in phospho-ERK levels from approximately 0.08-fold to 1.21-fold, compared to cells treated solely with corticosterone (*p* < 0.01). Furthermore, we employed the inhibitor U0126 to explore whether fisetin-induced CREB activation occurs through an ERK-dependent pathway. The data show that fisetin increased phospho-CREB levels by approximately 1.52-fold, and this effect remained unaltered by U0126 treatment in PC12 cells, where the levels increased by approximately 1.48-fold (Figure 7c,d). These results collectively indicate that fisetin induces CREB phosphorylation through a pathway that is independent of ERK activation.

### 3.6. Fisetin Increases FOXO3a Phosphorylation and Promotes Cytosolic Localization of FOXO3a in PC12 Cells

**Figure 8:**
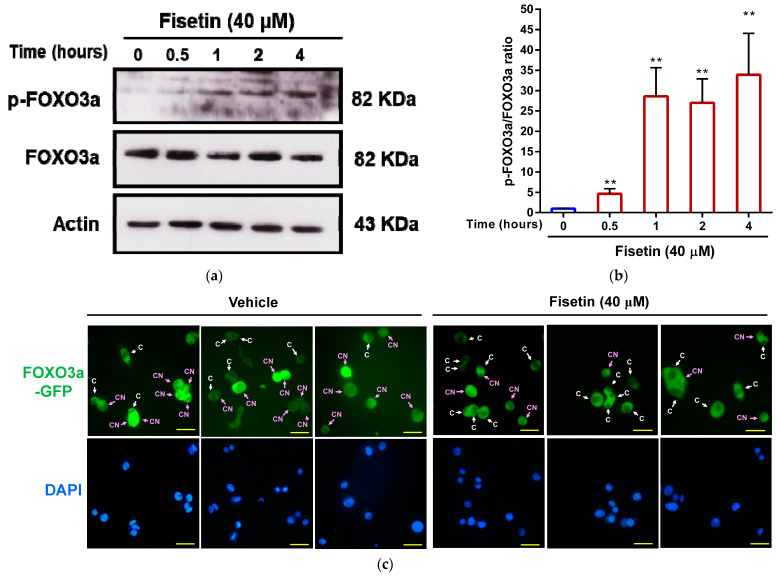
The effect of fisetin on the corticosterone-mediated retention of nuclear FOXO3a protein in PC12 cells. PC12 cells were treated with fisetin (40 μM) for 0, 0.5, 1, 2, and 4 h. (**a**) p-FOXO3a, FOXO3a, and actin proteins were measured by Western blot analysis, and a representative blot is shown. (**b**) Quantitative analysis of normalized intensity of p-FOXO3a versus FOXO3a. The data represent the mean ± SD of three independent experiments. ** *p* < 0.01 indicates significant differences compared to the 0 h group. (**c**) PC12 cells were cultured on a poly-L-lysine-coated coverslip, then transfected with pCMV6-AC-FOXO3 (GFP-tagged) plasmid for 24 h. The plasmid-transfected cells were treated with vehicle or fisetin (40 μM) for 24 h. After incubation, the cells were stained with DAPI. The FOXO3a-GFP protein (green) expressed in cells and the nuclei stained with DAPI (blue) were observed and photographed using fluorescence microscopy. The arrows showed the FOXO3a-GFP proteins located in the cytoplasm (C) and both in the cytoplasm and nucleus (CN). The representative images are shown, and the scale bar is 20 μm.

FOXO3a functions as a transcription factor, and its phosphorylation triggers translocation from the nucleus to the cytosol, subsequently downregulating the expression of genes linked to cell death and promoting cell survival [41]. As a result, we investigated the impact of fisetin on FOXO3a phosphorylation in PC12 cells using Western blot analysis. As depicted in Figure 8a,b, the protein ratio of phospho-FOXO3a (p-FOXO3a) to FOXO3a showed a significant increase in cells treated with fisetin. Furthermore, we utilized PC12 cells transfected with the plasmid expressing FOXO3-GFP proteins to confirm the effect of fisetin on the modulation of FOXO3a’s subcellular localization. As shown in Figure 8c, administering fisetin increased the cytosolic localization of FOXO3a-GFP proteins in PC12 cells compared to the vehicle-treated cells. These findings suggest that fisetin may promote FOXO3a phosphorylation, subsequently causing its translocation from the nucleus to the cytoplasm in neuronal cells.

### 3.7. Fisetin Counteracts the Corticosterone-Induced Nuclear Accumulation of FOXO3a through the PI3K/Akt Pathway

**Figure 9:**
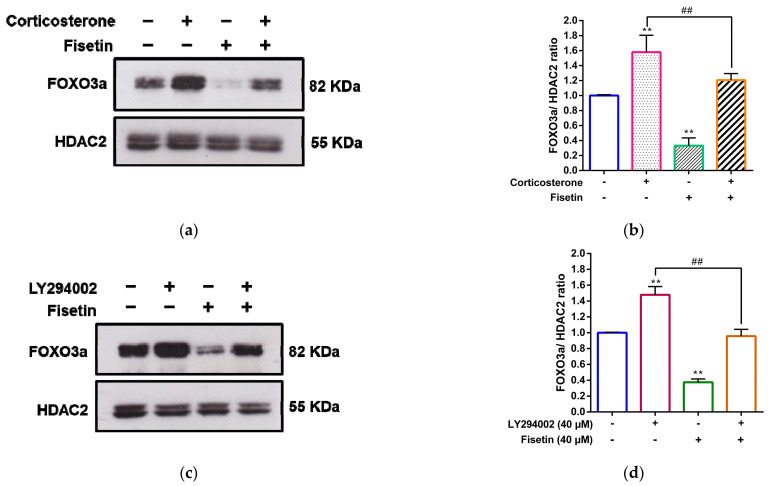
Fisetin counteracts the corticosterone-mediated retention of nuclear FOXO3a protein via the PI3K/Akt pathway. PC12 cells were treated with corticosterone (300 μM) in the absence or presence of fisetin (40 μM) for 24 h. (**a**) FOXO3a and HDAC2 proteins in the nucleus were measured by Western blot analysis, and a representative blot is shown. (**b**) A quantitative analysis of normalized intensity of FOXO3a versus HDAC2. The data represent the mean ± SD of three independent experiments. ** *p* < 0.01 indicates significant differences compared to the vehicle-treated group. ## *p* < 0.01 indicates significant differences compared to the corticosterone alone-treated group. PC12 cells were pre-treated with LY294002 (40 μM) for 30 min, then treated with fisetin (40 μM) for 24 h. (**c**) FOXO3a and HDAC2 proteins were measured by Western blot analysis, and a representative blot is shown. (**d**) Quantitative analysis of normalized intensity of FOXO3a versus HDAC2. The data represent the mean ± SD of three independent experiments. ** *p* < 0.01 indicates statistically significant differences compared to the vehicle-treated group. ## *p* < 0.01 indicates significant differences compared to the LY294002 alone-treated group.

To investigate the modulation of FOXO3a’s subcellular localization in corticosterone-treated cells, we performed a Western blot analysis to quantify nuclear FOXO3a protein levels in PC12 cells subjected to various treatments involving corticosterone, fisetin, or their combination. As illustrated in Figure 9a,b, corticosterone treatment significantly increased nuclear FOXO3a protein levels by approximately 1.58-fold compared to the vehicle-treated cells (*p* < 0.01). Conversely, fisetin treatment alone markedly reduced nuclear FOXO3a accumulation by approximately 0.33-fold (*p* < 0.01) in PC12 cells. Notably, in the co-treatment scenario involving corticosterone and fisetin, fisetin effectively attenuated the nuclear FOXO3a levels from around 1.58-fold to 1.21-fold (*p* < 0.01) when compared to cells exclusively treated with corticosterone. These results indicated that fisetin treatment promoted a pronounced translocation of FOXO3a proteins to the cytoplasm, contrasting with cells treated solely with corticosterone. Collectively, these findings reveal that fisetin significantly counteracts the corticosterone-induced nuclear retention of FOXO3a protein in PC12 cells.

Previous research has indicated that the activation of the PI3/Akt signaling pathway leads to the phosphorylation of FOXO3a, thus preventing the nuclear retention of FOXO3a [45]. We further investigated the effect of fisetin on the regulation of the PI3/Akt-FOXO3a pathway in corticosterone-treated PC12 cells. To explore this aspect, the experiments were initiated by pre-treating PC12 cells with LY294002, followed by their exposure to corticosterone and fisetin. The results, as shown in Figure 9c,d, demonstrated that the application of the inhibitor LY294002 led to an increase in nuclear FOXO3a levels within fisetin-treated cells. This observation suggests that fisetin possesses the capability to induce the cytoplasmic translocation of FOXO3a by activating the PI3/Akt pathway in corticosterone-treated cells. These results suggest fisetin’s potential in promoting cell survival against corticosterone-induced cell death, achieved through the orchestrated modulation of the PI3/Akt-FOXO3a pathway in PC12 cells.

## 4. Discussion

**Figure 10:**
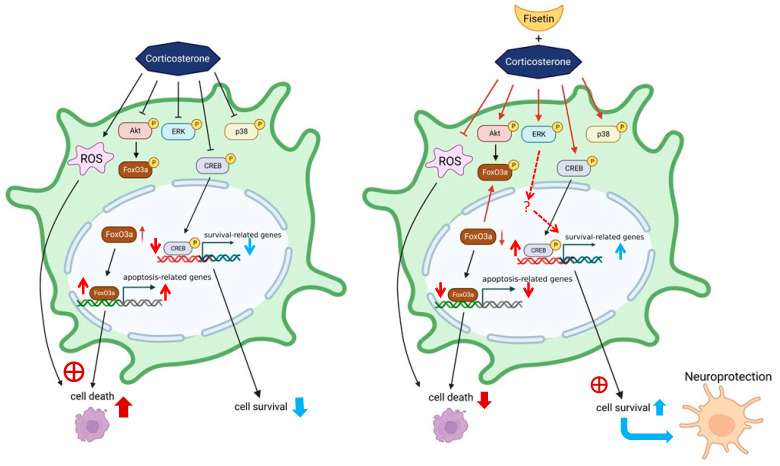
Hypothetical mechanism of the neuroprotective effect of fisetin against corticosterone-induced neuronal cell death. Fisetin has been shown to mitigate the corticosterone-mediated production of ROS and has the potential to inhibit apoptosis induced by corticosterone. Furthermore, fisetin demonstrates the capability to enhance cell survival and provide protection against corticosterone-induced cell death by modulating various survival pathways, including MAPK/ERK, p38 MAPK, CREB, and PI3K/Akt/FOXO3a, in PC12 cells. Thus, fisetin exerts a neuroprotective effect against corticosterone-induced neuronal cell death. (Created with BioRender.com).

In the context of this study, the flavonoid fisetin demonstrates its neuroprotective potential against corticosterone-induced cell death in PC12 cells. Fisetin exhibits the ability to potentially attenuate corticosterone-triggered ROS production and apoptosis in cells. Through the activation of ERK, p38, and CREB phosphorylation, fisetin enhances cell survival and safeguards against neuronal cell death caused by corticosterone. Furthermore, fisetin activates the PI3K/Akt pathway, leading to enhanced FOXO3a phosphorylation and cytoplasmic translocation, thereby decreasing the nuclear FOXO3a accumulation and preventing cell apoptosis prompted by corticosterone in PC12 cells (Figure 10). The PC12 cell line is recognized for displaying various typical neuronal characteristics and is extensively utilized in studies related to neurotoxicity and neuroprotection. Moreover, it is known for its ability to express a high level of glucocorticoid receptors. Due to these attributes, the PC12 cell line is widely employed as a model cell line in the development of neuroprotective agents [46,47]. Our current findings suggest that the neuroprotective efficacy of fisetin against corticosterone-induced neurotoxicity, combined with its ability to traverse the blood–brain barrier (BBB), holds the potential for it to emerge as a novel neuroprotective agent.

It is established that corticosterone can trigger cellular damage in PC12 cells. Moreover, corticosterone has been associated with the induction of depression-like behaviors [48], alterations in brain anatomy, including the inhibition of structural plasticity in hippocampal neurons in animal models [49]. In this present study, we substantiated that corticosterone significantly induced cytotoxic effects on the neuronal PC12 cell line in a dose-dependent manner. Phytochemicals encompass bioactive chemical compounds derived from plants, such as polyphenols and flavonoids, which are abundant in fruits, vegetables, herbs, and Chinese medicines [50,51]. Numerous studies underscore the multifaceted beneficial effects of phytochemicals, including antioxidant, anti-inflammatory, anti-cancer, and neuroprotective activities [51,52]. Research has indicated that certain dietary phytochemicals, like polyphenols and flavonoids, can effectively traverse the BBB, enhancing their potential as agents for the prevention or treatment of neurological disorders [53,54]. In prior investigations conducted by our research group, it was demonstrated that various phytochemicals, including fisetin [36], 5-demethylnobiletin [55], and luteolin [56], exhibited the capacity to stimulate neuritogenesis and provide neuroprotection in PC12 cells. In this study, we present evidence showcasing the significant ability of fisetin to counteract corticosterone-induced ROS generation and cell apoptosis, thereby promoting cell survival within PC12 cell models. Previous reports have demonstrated that fisetin prevents dexamethasone-induced cytotoxicity by attenuating ROS levels and apoptosis in hippocampal HT22 cell lines [57]. These findings suggest that fisetin could protect neuronal cells against corticosteroid-induced oxidative damage. It has been reported that high stress-induced releases of glucocorticoids can affect synaptic plasticity and the neuronal network in the hippocampus, impairing cognitive functions [58,59]. Gite et al. reported that fisetin could elevate brain-derived neurotrophic factor (BDNF) expression in corticosterone-treated human neuroblastoma cell lines [60]. BDNF is a neurotrophin in the brain that regulates neuronal development, synaptic plasticity, and neuroprotection, playing essential roles in cognition and memory [61]. These findings suggest that fisetin may modulate neuronal cell plasticity in the brain under corticosteroid exposure. Our current findings support the notion that dietary fisetin has the potential to act as a neuroprotective agent, preserving neuronal cell survival and regulating neuronal plasticity against corticosteroid-induced neurotoxicity in the brain.

It has been reported that intracellular kinase signaling cascades are considered responsible for promoting neuronal survival [62]. In this study, we demonstrated that fisetin significantly enhances the phosphorylation of ERK, p38, and Akt, leading to increased cell survival in corticosterone-treated PC12 cells. The MAPK/ERK pathway is known to play a vital role in regulating cell survival and proliferation [63]. In this study, fisetin was able to enhance the phosphorylation level of ERK1/2, in concordance with the results from our previous study [36,64]. Additionally, the p38 MAPK pathway plays a significant role in cell differentiation and regulating cell survival against oxidative stress [65]. Furthermore, it may contribute to inflammation, apoptosis, and cell cycle regulation [66]. Akt phosphorylation has been reported to regulate neuronal toxicity through various substrates, including FOXOs and GSK3β, among others [67]. These findings support the notion that fisetin modulates the MAPK and PI3K/Akt pathways, thereby offering protection to neuronal cells against corticosterone-induced cell death, which could potentially contribute to the prevention or treatment of corticosteroids-induced neurotoxicity.

CREB is a transcription factor that is involved in numerous functions within neuronal cells, including the promotion of neuronal cell survival, neuronal differentiation, migration, and synaptogenesis [68]. In this study, our results revealed that fisetin significantly elevates the phosphorylation level of CREB, thereby effectively reversing the corticosterone-induced inactivation of CREB. This restoration of CREB activity is associated with enhanced neuronal cell survival. In our results, fisetin not only reversed the corticosterone-mediated inactivation of CREB, but also countered the corticosterone-induced inactivation of ERK. Nevertheless, our results demonstrated that the elevation of phosphorylated CREB levels induced by fisetin remained unaffected by the MAPK/ERK inhibitor U0126. This suggests that CREB might not be directly modulated by fisetin-mediated ERK activation in PC12 cells. As previously mentioned, the phosphorylation of the CREB protein can also be facilitated by the PI3K/Akt kinase. Our results demonstrated that fisetin could indeed enhance the phosphorylation of Akt. However, whether fisetin-induced CREB activation occurs through a PI3K/Akt-dependent pathway requires further investigation.

It has been reported that the activity of FOXO3a can be regulated through post-translational modifications, including phosphorylation, methylation, acetylation, and ubiquitination. The post-translational phosphorylation of the FOXO3a protein influences its localization within various cellular compartments, ultimately determining whether it becomes inactivated (in the cytoplasm) or activated (in the nucleus) [45,69]. In this study, fisetin treatment increased the levels of FOXO3a phosphorylation and induced the cytoplasmic localization of FOXO3a through a PI3K/Akt-dependent pathway in PC12 cells. Brunet et al. reported that PI3K/Akt activation enhances cell survival by phosphorylating and inhibiting the activity of the FOXO3a protein [70]. Previous studies have shown that activated FOXO3a can induce the expression of downstream death-related genes, such as the p53-upregulated modulator of apoptosis (Puma) and Bcl-2-interacting mediator of cell death (Bim) [41]. In our study, we observed an increased amount of FOXO3a in the nucleus, alterations in cell morphology, and cell death due to corticosterone treatment. Our current findings support the notion that fisetin-mediated neuroprotection against corticosterone-induced cell death may be correlated with the phosphorylation of FOXO3a through the PI3K/Akt pathway in neuronal cells. However, the specific signaling molecules involved in fisetin-mediated FOXO3a translocation remain unclear and require further investigation.

In conclusion, our findings suggest that fisetin exerts a neuroprotective effect against corticosterone-induced cell death through multiple mechanisms, including ROS reduction, apoptosis inhibition, and the promotion of cell survival. These effects are achieved through the modulation of various pathways such as MAPK/ERK-, p38-, and the PI3/Akt/FOXO3a-dependent pathways. However, it is important to note that the PC12 cell lines used in this study may not perfectly reflect the complexities of neuronal cells. Given these promising results, further investigation is warranted to fully understand the neuroprotective effects of fisetin in primary neurons. This should include animal and human studies to better evaluate the potential preventive or therapeutic benefits of fisetin in corticosteroid-induced neuronal or psychiatric disorders.

## Data Availability

The data can be shared up on request.
